# Efficacy of Twice a Day Bismuth Quadruple Therapy for Second-Line Treatment of *Helicobacter pylori* Infection

**DOI:** 10.3390/jpm12010056

**Published:** 2022-01-06

**Authors:** Jeemyoung Kim, Eun Jeong Gong, Myeongsook Seo, Hyun Il Seo, Jong Kyu Park, Sang Jin Lee, Koon Hee Han, Woo Jin Jeong, Young Don Kim, Gab Jin Cheon

**Affiliations:** Department of Internal Medicine, Gangneung Asan Hospital, University of Ulsan College of Medicine, Gangneung 25440, Korea; kjm690@naver.com (J.K.); msseo.md@gmail.com (M.S.); reshi@hanmail.net (H.I.S.); sajahoooo@naver.com (J.K.P.); sangjin@naver.com (S.J.L.); hankoonhee@hanmail.net (K.H.H.); jwoojini@naver.com (W.J.J.); ydkim@gnah.co.kr (Y.D.K.); 1000@gnah.co.kr (G.J.C.)

**Keywords:** drug administration schedule, *Helicobacter pylori*, treatment outcome

## Abstract

Bismuth quadruple therapy (BQT) is an effective treatment for *Helicobacter pylori* infection. However, frequent dosing schedules of BQT regimen often compromise drug adherence and may affect treatment outcomes. This retrospective study aimed to investigate the efficacy of twice-daily BQT compared to that of four times a day therapy. From August 2018 to November 2020, adult patients who failed first-line standard triple therapy and underwent BQT were eligible. Patients were categorized into two groups according to dosing schedule: (i) the BQT group (*n* = 213) who received standard BQT administered four times a day; and (ii) the BQTb group (*n* = 141) who received proton pump inhibitor, bismuth 600 mg, metronidazole 500 mg, and tetracycline 1 g twice a day. The eradication rate did not differ between the BQT (92.5%) and the BQTb groups (90.1%) (*p* = 0.441). Adherence and adverse event rate were similar between the two groups. Multivariate analysis showed that current smoking was associated with eradication failure; however, dosing frequency was not associated with the efficacy of eradication therapy. This study suggested that twice a day BQT is as effective as four times a day therapy for second-line treatment of *H. pylori* infection.

## 1. Introduction

*Helicobacter pylori* infection is known to be associated with the development of peptic ulcer disease, gastric cancer, and gastric mucosa-associated lymphoid tissue lymphoma [1]. In addition, associations between *H. pylori* infection and extra-gastrointestinal conditions, such as unexplained iron deficiency anemia and immune thrombocytopenia, have been suggested [2,3]. Indication for *H. pylori* eradication therapy has been expanded and current guidelines and consensus reports recommend *H. pylori* eradication therapy for the treatment of these conditions [2,3,4,5,6,7].

Recent guideline for the treatment of *H. pylori* infection in Korea recommends bismuth quadruple therapy (BQT) consisting of a proton pump inhibitor (PPI), bismuth, metronidazole, and tetracycline as a second-line therapy after failure of standard triple therapy (STT) or sequential therapy, and as a first-line therapy when clarithromycin resistance is suspected [3]. Although BQT is an effective eradication regimen, frequent dosing may make it uncomfortable for patients to adhere to the treatment schedule. However, in addition to an optimal dose of bismuth and antimicrobial agents, the appropriate dosing schedule of BQT and its impact on the effectiveness of *H. pylori* eradication therapy remain unclear.

Some researchers have investigated the efficacy of twice a day dosing of BQT and showed promising results in terms of a high eradication rate of over 90% [8,9,10]. However, they were not comparative studies [9,10] or used antimicrobial agents at doses different from that of standard BQT [8]. In this study, we compared the efficacy of twice-daily BQT with that of four times a day regimen and investigated factors associated with failure of eradication therapy in a single tertiary care hospital.

## 2. Materials and Methods

### 2.1. Study Population

**Figure 1:**
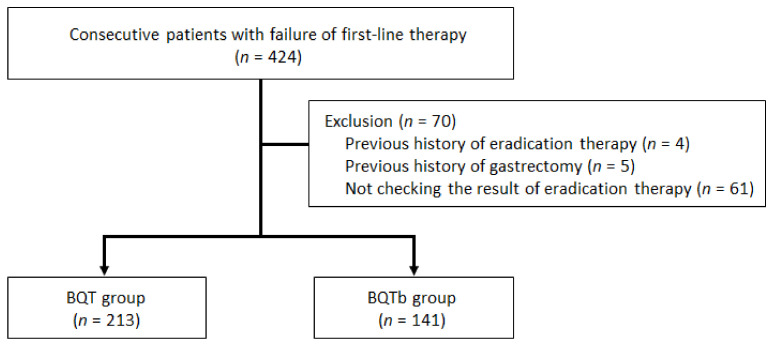
Flow chart of the study. BQT: conventional bismuth quadruple therapy; BQTb: twice a day BQT.

From August 2018 to November 2020, consecutive patients who underwent BQT for 7 to 14 days after the failure of first-line STT were eligible. Of the 424 patients, 4 patients with a history of previous eradication therapy, 5 patients who had received gastrectomy, and 61 patients who did not check the result of eradication therapy were excluded from the study. Finally, a total of 354 patients were included in the analysis (Figure 1). Data regarding demographic factors—such as age, gender, comorbidities, history of smoking, and habitual consumption of alcohol—and therapy-related characteristics were collected using medical records. The study protocol was approved by the Institutional Review Board (No, 2021-11-001), and written informed consent was waived.

### 2.2. Treatment and Follow-Up

Patients were divided into two groups according to dosing schedule: (i) the BQT group (*n* = 213) who received standard BQT consisting of PPI twice a day, bismuth subcitrate (DeNol) 300 mg four times a day, metronidazole 500 mg three times a day, and tetracycline 500 mg four times a day; and (ii) the BQTb group (*n* = 141) who received PPI, bismuth subcitrate 600 mg, metronidazole 500 mg, and tetracycline 1 g twice a day. The choice of treatment regimen and duration were determined based on the preference of the physician. Results of eradication therapy were assessed four weeks or more after completion using ^14^C-urea breath test or histologic examination by modified Giemsa staining of biopsy tissue.

### 2.3. Statistical Analysis

Continuous variables are shown as median (range), and categorical variables are shown as numbers (percentage). Differences in baseline characteristics were tested using the chi-square test, Fisher’s exact test, *t*-test, or Mann–Whitney *U*-test, as appropriate. A logistic regression model was used to identify the factors associated with eradication failure, and odds ratio (OR) and 95% confidence interval (CI) were estimated. All statistical analyses were performed using SPSS version 24.0 (SPSS Inc., Chicago, IL, USA).

## 3. Results

### 3.1. Study Population

The baseline characteristics of the study population are summarized in Table 1. The median age of 354 patients was 59 years (range, 20–81 years) and 51.7% were male. Regarding smoking history, one-third of the patients were previous or current smokers. There was no significant difference in age, underlying disorders, and smoking and drinking habits between the two groups.

Table 2 shows the characteristics associated with eradication therapy. Atrophic gastritis was the most common indication of eradication therapy (44.9%) in both groups, followed by peptic ulcer disease (26.8%), and others (15.8%) such as gastric adenoma after endoscopic resection, gastric polyp, and lymphofollicular gastritis. Most patients (83.9%) received 14-day course of eradication therapy, followed by 10 days (11.0%) and 7 days (5.1%).

### 3.2. Results of Eradication Therapy

**Figure 2:**
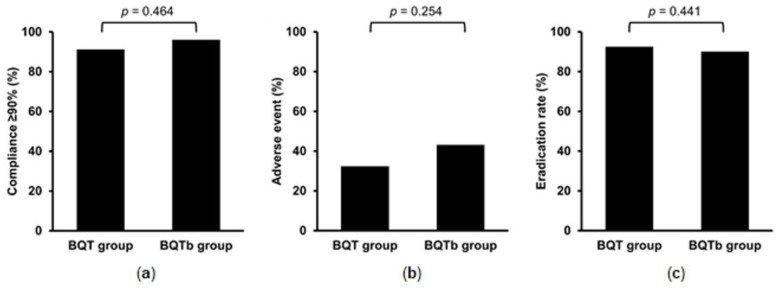
Comparison of factors associated with eradication therapy. (**a**) The proportion of patients with adherence ≥90% (*n* = 119); (**b**) adverse event rates (*n* = 119); (**c**) eradication rate for bismuth quadruple therapy four times a day (BQT group) and twice a day bismuth quadruple therapy (BQTb group).

The overall eradication rate was 91.5% (324/354). The eradication rate did not differ between the BQT and BQTb groups (92.5% and 90.1%, *p* = 0.441) (Figure 2). Adherence and the presence of adverse events could have been evaluated in 119 patients. The proportion of patients who took equal or greater than 90% of the medication was 91.2% in BQT group and 96.1% in BQTb group (*p* = 0.464). Adverse events were encountered by 37.0% of patients, including gastrointestinal symptoms such as abdominal discomfort and nausea. Adverse event rates were similar between the two groups (32.4% and 43.1%, *p* = 0.254).

### 3.3. Factors Associated with Eradication Failure

Among the various characteristics, current smoking was significantly related to eradication failure in univariate analysis (OR 2.969, 95% CI 1.230 to 7.167, *p* = 0.015) and multivariate analysis (OR 3.029, 95% CI 1.077 to 8.521, *p* = 0.036) (Table 3). There was no significant association between eradication failure and other factors, such as sex and dosing frequency.

## 4. Discussion

In this study, we investigated the efficacy of twice a day BQT as second-line therapy for *H. pylori* eradication. The eradication rate did not differ between the BQT and BQTb groups. There was no significant difference in the drug adherence and adverse event related to therapy between the two groups. Logistic regression analysis showed that BQT dosing frequency was not associated with eradication failure, but current smoking was a factor that was significantly related to eradication failure.

Increase in antibiotic resistance and decrease in eradication rate are emerging global issues in *H. pylori* eradication therapy [11]. In Korea, the same tendency was found in some studies of patients who received clarithromycin-based STT [12,13]. Indeed, the overall resistance rate against clarithromycin was reported as 17.8% in the recent nationwide study in Korea [14], and the reported overall eradication rate of STT was 71.7% in the nationwide registry study [15]. Although there is a geographic difference, a resistance rate of more than 15% against clarithromycin is generally considered high, and STT as an empirical treatment is no longer recommended in areas with a high probability of clarithromycin resistance. BQT or concomitant therapy may be a suitable treatment option in this situation; however, quadruple therapy has a drawback of exposing patients to one or more unnecessary antibiotics.

Optimal eradication regimen for *H. pylori* infection is evolving, and the latest guidelines and consensus reports refer to eradication therapy based on the result of culture-based antibiotic susceptibility test [2,3,4,5,6,7]. Such tailored therapy is ideally desirable; however, it is difficult to apply in clinical practice for several reasons [16]. First, the investigated success rate of *H. pylori* culture from gastric biopsies was as low as 55–73%. Second, a bacterial culture is time-consuming and requires a specific environment. Third, the growth of *H. pylori* can be affected by many factors such as the number of specimens, the culture conditions, and gastrointestinal bleeding. Moreover, the result from biopsied samples may not represent the conditions of the entire stomach [17,18].

BQT is an effective second-line or salvage therapy after one or more eradication failures [13,19,20]. However, a conventional BQT regimen involves taking medications four times a day during the entire period of eradication therapy. The complicated and uncomfortable features of this regimen could be an obstacle to achieving better adherence and treatment success. Several studies have evaluated the efficacy of twice-daily BQT and showed an eradication rate of 90% or higher [8,9,10]. However, these studies were not comparative [8,9] or used antimicrobial agents and bismuth at modified doses [10]. In the present study, we compared the efficacy of twice a day BQT regimen with that of conventional BQT as second-line eradication therapy. The eradication rate of twice a day BQT regimen was 90.1% and was similar to that of conventional BQT, which was given four times a day. The observed eradication rate of BQT was consistent with the recently reported annual eradication rate of second-line BQT in Korea [19], suggesting a possibility of decreasing the dosing frequency of BQT while maintaining efficacy.

In determining whether the twice a day BQT can be generalized, the doses and treatment duration of antimicrobial agents and bismuth should be carefully considered. It is recommended to use at least 1500 mg of metronidazole to overcome antimicrobial resistance to metronidazole [20]. In this study, the eradication rate was higher than expected for twice a day BQT with a relatively lower dose (1000 mg/day) of metronidazole, considering the high resistance rate against metronidazole in Korea [14,19]. It is presumably attributed to the long duration of therapy in majority of the patients and a sufficient dose of bismuth, which have additive effects on eradication rates despite high metronidazole resistance [21]. Regional data on the prevalence of antimicrobial resistance of *H. pylori* against metronidazole are necessary to support this speculation.

Several factors—including sex, smoking status, and *CYP2C19* polymorphism—have been reported to be associated with eradication failure [12,22,23,24,25]. In the present study, current smoking showed a significant association with eradication failure, while other factors such as sex and dosing frequency did not appear to be associated with treatment failure. This result is in line with previous findings that smoking increases the likelihood of eradication failure [12,22,24,25]. The presumed mechanisms for explaining the impact of smoking on eradication failure include decreased gastric blood flow and mucous secretion, more acidic environment, and tendency of poor adherence by smokers [12,22,25]. Although it was statistically insignificant in this study, females more frequently experience eradication failure [12]. It was suggested that women infected with *H. pylori* preferentially carry the *A2143G* mutation in the 23S rRNA, which affects the failure of clarithromycin-based STT. Another possible factor affecting eradication failure is *CYP2C19* genetic polymorphism. Because most PPIs are predominantly metabolized through CYP2C19 pathway, the pharmacologic effect of PPIs can be affected by *CYP2C19* genetic polymorphism [23], and the altered effects of PPIs can affect the efficacy of eradication therapy. Duration of eradication therapy also affects treatment outcomes. Theoretically, a longer treatment duration helps PPIs to reach their full anti-secretory effects in eradicating *H. pylori* of different niches [26]. In addition, BQT with increased metronidazole dosage and duration extended to 14 days was associated with eradication rates of greater than 90% [16]. Indeed, in a previous study comparing the efficacy of second-line BQT according to treatment duration, the 14-day BQT group showed a significantly higher eradication rate than the 7-day group [3].

This study has several limitations. Firstly, being a retrospective study, some of the clinical data were incomplete. Primarily, drug adherence and adverse event data were obtained based on the medical records. Secondly, an antimicrobial susceptibility test was not performed. Thirdly, this is the result of a single-center study. Considering differences in geographic and demographic features, which could affect the clinical courses and treatment outcomes associated *H. pylori* infection, the results of the present study cannot be generalized. A future prospective study including antimicrobial susceptibility testing would be beneficial.

## 5. Conclusions

In conclusion, twice-daily BQT regimen was as effective as conventional four times a day BQT regimen after the failure first-line STT. In addition to the antimicrobial resistance, failure to adhere to the complex multidrug eradication regimen is one of important reasons for eradication failure. Therefore, a twice-daily BQT regimen may be a promising treatment option to improve patients’ adherence while maintaining the eradication rate.

## Figures and Tables

**Table 1:** Demographic and clinical characteristics of the study population.

	Total (*n* = 354)	BQT Group (*n* = 213)	BQTb Group (*n* = 141)	*p*-Value
Age, years	59 (20–81)	58 (20–79)	60 (36–81)	0.711
Male	183 (51.7)	105 (49.3)	78 (55.3)	0.279
Comorbidities				
Hypertension	112 (31.6)	67 (31.5)	45 (31.9)	1.000
Diabetes	55 (15.5)	38 (17.8)	17 (12.1)	0.177
Chronic kidney disease	1 (0.3)	1 (0.5)	0 (0.0)	1.000
Liver cirrhosis	1 (0.3)	1 (0.5)	0 (0.0)	1.000
Coronary artery disease	8 (2.3)	5 (2.3)	3 (2.1)	1.000
Cerebrovascular accident	9 (2.5)	3 (1.4)	6 (4.3)	0.164
Smoking (*n* = 311)				0.341
Never	206 (66.2)	118 (65.6)	88 (67.2)	
Previous	45 (14.5)	23 (12.8)	22 (16.8)	
Current	60 (19.3)	39 (21.7)	21 (16.0)	
Alcohol consumption (*n* = 312)				0.347
Never	159 (51.0)	93 (51.4)	66 (50.4)	
Previous	7 (2.2)	6 (3.3)	1 (0.8)	
Current	146 (46.8)	82 (45.3)	64 (48.9)	

Results are reported as number (%), except for age, which is reported as median (range). BQT, bismuth quadruple therapy; BQTb, twice-a-day BQT regimen.

**Table 2:** Characteristics associated with eradication therapy.

	**Total** **(*n* = 354)**	**BQT Group** **(*n* = 213)**	**BQTb Group** **(*n* = 141)**	***p*-Value**
Indication				0.028
Peptic ulcer disease	95 (26.8)	57 (26.8)	38 (27.0)	
MALT lymphoma	3 (0.8)	1 (0.5)	2 (1.4)	
Early gastric cancer	15 (4.2)	12 (5.6)	3 (2.1)	
Dyspepsia	19 (5.4)	9 (4.2)	10 (7.1)	
Family history	7 (2.0)	4 (1.9)	3 (2.1)	
Atrophic gastritis	159 (44.9)	87 (40.8)	72 (51.1)	
Others ^1^	56 (15.8)	43 (20.2)	13 (9.2)	
Duration of treatment				<0.001
7 days	18 (5.1)	17 (8.0)	1 (0.7)	
10 days	39 (11.0)	36 (16.9)	3 (2.1)	
14 days	297 (83.9)	160 (75.1)	137 (97.2)	
Use of supplementary agents				<0.001
None	303 (85.6)	211 (99.1)	92 (65.2)	
Mucoprotective agent	1 (0.3)	1 (0.5)	0 (0.0)	
Probiotics	50 (14.1)	1 (0.5)	49 (34.8)	
Adherence ≥90% ^2^	111 (93.3)	62 (91.2)	49 (96.1)	0.464
Adverse event ^2^	44 (37.0)	22 (32.4)	22 (43.1)	0.254
Eradication rate	324 (91.5)	197 (92.5)	127 (90.1)	0.441

Results are reported as number (%). ^1^ Including gastric adenoma after endoscopic resection, gastric polyp, and lymphofollicular gastritis. ^2^ Data on adherence and adverse event were available for 119 patients. BQT, bismuth quadruple therapy; BQTb, twice a day BQT regimen; MALT, mucosa-associated lymphoid tissue.

**Table 3:** Factors associated with eradication failure.

	Eradication Failure/Total	Univariate Analysis		Multivariate Analysis	
		OR (95% CI)	*p-*Value	OR (95% CI)	*p-*Value
Sex					
Male	20/183	Reference			
Female	10/171	0.506 (0.230–1.115)	0.091	0.968 (0.353–2.656)	0.950
Smoking					
Nonsmoker	13/206	Reference		Reference	
Ex-smoker	5/45	1.856 (0.626–5.498)	0.265	1.775 (0.501–6.292)	0.374
Current smoker	10/60	2.969 (1.230–7.167)	0.015	3.029 (1.077–8.521)	0.036
Duration, days					
7 days	1/18	Reference			
10 days	3/39	0.613 (0.078–4.794)	0.641		
14 days	26/297	0.869 (0.250–3.015)	0.824		
Use of supplementary agents					
No	23/303	Reference			
Yes	7/51	1.937 (0.784–4.782)	0.152		
Adherence					
≥90	14/111	Reference			
<90%	2/8	2.310 (0.424–12.586)	0.333		
Dosing frequency					
Four times a day	16/213	Reference			
Twice a day	14/141	1.357 (0.640–2.877)	0.425	1.506 (0.681–3.329)	0.312

CI, confidence interval; OR, odds ratio.

## Data Availability

Data available upon request to corresponding author.

## References

[1] McColl K.E.L. (2010). Helicobacter pyloriInfection. N. Engl. J. Med..

[2] Malfertheiner P., Megraud F., O’Morain C.A., Gisbert J.P., Kuipers E.J., Axon A.T., Bazzoli F., Gasbarrini A., Atherton J., Graham D.Y. (2017). Management of Helicobacter pylori infection—the Maastricht V/Florence Consensus Report. Gut.

[3] Jung H.-K., Kang S.J., Lee Y.C., Yang H.-J., Park S.-Y., Shin C.M., Kim S.E., Lim H.C., Kim J.-H., Nam S.Y. (2021). Evidence-Based Guidelines for the Treatment of Helicobacter pylori Infection in Korea 2020. Gut Liver.

[4] Fallone C.A., Chiba N., van Zanten S.V., Fischbach L., Gisbert J.P., Hunt R.H., Jones N.L., Render C., Leontiadis G.I., Moayyedi P. (2016). The Toronto Consensus for the Treatment of Helicobacter pylori Infection in Adults. Gastroenterology.

[5] Chey W.D., Leontiadis G.I., Howden C.W., Moss S.F. (2017). ACG Clinical Guideline: Treatment of Helicobacter pylori Infection. Am. J. Gastroenterol..

[6] Mahachai V., Vilaichone R.-K., Pittayanon R., Rojborwonwitaya J., Leelakusolvong S., Maneerattanaporn M., Chotivitayatarakorn P., Treeprasertsuk S., Kositchaiwat C., Pisespongsa P. (2017). Helicobacter pylori management in ASEAN: The Bangkok consensus report. J. Gastroenterol. Hepatol..

[7] Liou J.-M., Malfertheiner P., Lee Y.-C., Sheu B.-S., Sugano K., Cheng H.-C., Yeoh K.-G., Hsu P.-I., Goh K.-L., Mahachai V. (2020). Screening and eradication of Helicobacter pylori for gastric cancer prevention: The Taipei global consensus. Gut.

[8] Dore M., Graham D., Mele R., Marras L., Nieddu S., Manca A., Realdi G. (2002). Colloidal bismuth subcitrate-based twice-a-day quadruple therapy as primary or salvage therapy for Helicobacter pylori infection. Am. J. Gastroenterol..

[9] Dore M.P., Marras L., Maragkoudakis E., Nieddu S., Manca A., Graham D.Y., Realdi G. (2003). Salvage therapy after two or more prior Helicobacter pylori treatment failures: The super salvage regimen. Helicobacter.

[10] Kim J.Y., Lee S.-Y., Kim J.H., Sung I.-K., Park H.S. (2020). Efficacy and safety of twice a day, bismuth-containing quadruple therapy using high-dose tetracycline and metronidazole for second-line Helicobacter pylori eradication. Helicobacter.

[11] Thung I., Aramin H., Vavinskaya V., Gupta S., Park J.Y., Crowe S.E., Valasek M.A. (2016). Review article: The global emergence of *Helicobacter pylori* antibiotic resistance. Aliment. Pharmacol. Ther..

[12] Kim S.E., Park M.I., Park S.J., Moon W., Choi Y.J., Cheon J.H., Kwon H.J., Ku K.H., Yoo C.H., Kim J.H. (2015). Trends in Helicobacter pylori eradication rates by first-line triple therapy and related factors in eradication therapy. Korean J. Intern. Med..

[13] Shin W.G., Lee S.W., Baik G.H., Huh K.C., Chung J.-W., Jung W.T., Park M.I., Jung H.-K., Kim H.U., Kim J.H. (2015). Eradication Rates of Helicobacter pylori in Korea Over the Past 10 years and Correlation of the Amount of Antibiotics Use: Nationwide Survey. Helicobacter.

[14] Lee J.H., Ahn J.Y., Choi K.D., Jung H., Kim J.M., Baik G.H., Kim B., Park J.C., Jung H., Cho S.-J. (2019). Nationwide antibiotic resistance mapping of Helicobacter pylori in Korea: A prospective multicenter study. Helicobacter.

[15] Kim B.J., Yang C., Song H.J., Jeon S.W., Kim G.H., Kim H., Kim T.H., Shim K., Chung I., Park M.I. (2019). Online registry for nationwide database of Helicobacter pylori eradication in Korea: Correlation of antibiotic use density with eradication success. Helicobacter.

[16] Graham D.Y., Dore M.P. (2016). Helicobacter pylori therapy: A paradigm shift. Expert Rev. Anti Infect. Ther..

[17] Bilgilier C., Stadlmann A., Makristathis A., Thannesberger J., Kastner M.-T., Knoflach P., Steiner P., Schöniger-Hekele M., Högenauer C., Blesl A. (2018). Prospective multicentre clinical study on inter- and intrapatient genetic variability for antimicrobial resistance of Helicobacter pylori. Clin. Microbiol. Infect..

[18] Kocsmár É., Kocsmár I., Buzás G.M., Szirtes I., Wacha J., Takáts A., Hritz I., Schaff Z., Rugge M., Fassan M. (2019). Helicobacter pylori heteroresistance to clarithromycin in adults-New data by in situ detection and improved concept. Helicobacter.

[19] Yoon K., Kim N., Lee J.W., Yoon H., Shin C.M., Park Y.S., Lee D.H. (2020). Annual eradication rate of bismuth-containing quadruple therapy as second-line treatment for Helicobacter pylori infection: A 15-year prospective study at a tertiary hospital in Korea. Helicobacter.

[20] Graham D.Y., Lee S.-Y. (2015). How to Effectively Use Bismuth Quadruple Therapy: The Good, the Bad, and the Ugly. Gastroenterol. Clin. N. Am..

[21] Graham D.Y., Dore M.P., Lu H. (2018). Understanding treatment guidelines with bismuth and non-bismuth quadruple Helicobacter pylori eradication therapies. Expert Rev. Anti Infect. Ther..

[22] Itskoviz D., Boltin D., Leibovitzh H., Perets T.T., Comaneshter D., Cohen A., Niv Y., Levi Z. (2017). Smoking increases the likelihood of Helicobacter pylori treatment failure. Dig. Liver Dis..

[23] Kim J., Kim Y.-J., Chung W.C. (2020). Proton Pump Inhibitor Switching Strategy after Failure of Standard Triple Therapy for Helicobacter pylori Infection. Korean J. Helicobacter Up. Gastrointest. Res..

[24] Yu J., Yang P., Qin X., Li C., Lv Y., Wang X. (2021). Impact of smoking on the eradication of Helicobacter pylori. Helicobacter.

[25] Shin K., Cho M.-J., Oh J.-H., Lim C.-H. (2021). Second-Line Bismuth-Containing Quadruple Therapy for *Helicobacter pylori* Infection: A 12-Year Study of Annual Eradication Rates. J. Clin. Med..

[26] Shiotani A., Lu H., Dore M.P., Graham D.Y. (2017). Treating Helicobacter pylori effectively while minimizing misuse of antibiotics. Cleve. Clin. J. Med..

